# Validation of the Modified Berlin Questionnaire for the Diagnosis of Obstructive Sleep Apnea in Patients with a History of COVID-19 Infection

**DOI:** 10.3390/jcm12093047

**Published:** 2023-04-22

**Authors:** Yeliz Celik, Arzu Baygül, Yüksel Peker

**Affiliations:** 1 Graduate School of Health Sciences, Koc Universitesi, 34450 Istanbul, Turkey; abaygul@ku.edu.tr; 2 Research Center for Translational Medicine (KUTTAM), Koc University School of Medicine, 34450 Istanbul, Turkey; yuksel.peker@lungall.gu.se; 3 Irving Medical Center, Columbia Universitesi, New York, NY 10027, USA; 4 Department of Pulmonary Medicine, Koc University School of Medicine, 34450 Istanbul, Turkey; 5 Department of Molecular and Clinical Medicine, Institute of Medicine, Sahlgrenska Academy, University of Gothenburg, 41319 Gothenburg, Sweden; 6 Division of Sleep and Circadian Disorders, Brigham and Women’s Hospital, Boston, MA 02115, USA; 7 Division of Pulmonary, Allergy, and Critical Care Medicine, University of Pittsburgh School of Medicine, Pittsburgh, PA 15261, USA; 8 Department of Clinical Sciences, Respiratory Medicine and Allergology, Faculty of Medicine, Lund University, 22100 Lund, Sweden

**Keywords:** obstructive sleep apnea, COVID-19, Berlin questionnaire

## Abstract

(1) Background: The Berlin questionnaire (BQ) is a widely used survey to predict obstructive sleep apnea (OSA). Considering the confounding effect of obesity and hypertension on the clinical course of COVID-19, we have recently developed a modified BQ (mBQ) based on the subscales snoring intensity/frequency, witnessed apneas and morning/daytime tiredness, and demonstrated that patients with high-risk OSA had worse outcomes during the COVID-19 pandemic. In the current study, we aimed to validate the mBQ in adults with a history of COVID-19 infection. (2) Method: All cases who suffered from COVID-19 infection between 10 March and 22 June 2020, and who completed the mBQ in our first study, were invited to participate. Participants refilled the questionnaires, and an attended polysomnography (PSG) was conducted. An apnea−hypopnea index (AHI) of 15 events/h or more was considered as OSA. (3) Results: Out of the 70 participants, 27 (39%) were categorized as having a high risk of OSA based on the mBQ. According to the PSG results, 24 patients with high-risk OSA (89%) and 3 patients with low-risk OSA on the mBQ (7%) had AHI ≥ 15 events/h. The mBQ had a sensitivity of 89%, a specificity of 93%, a positive predictive value of 89%, a negative predictive value of 93%, and an accuracy of 91%. The area under the curve was 0.91 confirming a very good performance of the mBQ in screening for OSA. (4) Conclusions: The mBQ has a good level of diagnostic sensitivity, specificity, and accuracy among adults with a history of COVID-19 infection. Since the confounding effects of obesity and hypertension are eliminated, the mBQ may be used not only as a screening tool for high-risk OSA but also as a prognostic survey in clinical cohorts.

## 1. Introduction

The coronavirus disease 2019 (COVID-19) has caused a major public health crisis worldwide and critically affected the lives of millions of people since the first cases were reported from Wuhan, China, in December 2019. According to the estimates of the World Health Organization, as of 16 March 2022, 761 million people were infected with COVID-19, and more than 6, 8 million people died globally. 

Hypertension, diabetes, and cardiovascular diseases were identified as the most common comorbid diseases in patients with a history of COVID-19 infection [1,2,3,4]. During the first months of the pandemic, research letters and brief reports have suggested that individuals with OSA might be more susceptible to COVID-19 infection and might have a worse clinical course than those without OSA [5,6,7]. In retrospective analyses of medical records, a known OSA diagnosis in COVID-19 cases was reported to be 10 to 12% [3,8]. In the article published by Cade et al. [8], it has been shown that the mortality rate in COVID-19 cases with a known OSA diagnosis is higher than the controls. The coronavirus SARS-CoV-2 and diabetes outcomes (CORONADO) study suggested that patients with diabetes who were hospitalized for COVID-19 had an almost three-fold increased risk of death on day 7 [4]. 

There is no information yet about the actual prevalence of OSA in patients with COVID-19 infection, since objective sleep studies with polysomnography (PSG) were not feasible during an active contagious respiratory infection. In this context, one approach could be the use of surveys for estimating the prevalence of OSA in patients with COVID-19 infection, which has been the rationale of our previous study [9]. Given that the Berlin questionnaire (BQ) is a widely used survey to predict OSA, we conducted a prospective observational cohort study among 320 patients diagnosed with COVID-19 between 10 March and 22 June 2020, and estimated the prevalence of high-risk OSA as 37.8% based on the BQ [9]. Although the BQ has been widely used as a screening tool for OSA in general populations [10] and clinical cohorts [11], it has yet not been validated in patients with COVID-19 infection. Since obesity and hypertension are known to have adverse effects on the clinical course of COVID-19, we have also modified the BQ scoring system by ignoring these conditions in order to better determine the prognostic role of high-risk OSA in patients with a history of COVID-19 infection [9]. The estimated OSA prevalence was 21.9% based on the modified BQ (mBQ) when obesity and hypertension were excluded. Our results suggested that the patients with modified high-risk OSA had poorer clinical outcomes compared with those with modified low-risk OSA independent of gender, age, and comorbidities [9]. 

In the current study, we aimed to validate the mBQ among patients with a history of COVID-19 infection, who participated in the initial study.

## 2. Materials and Methods

### 2.1. Study Design, Participants, and Ethics Approval

**Figure A2:**
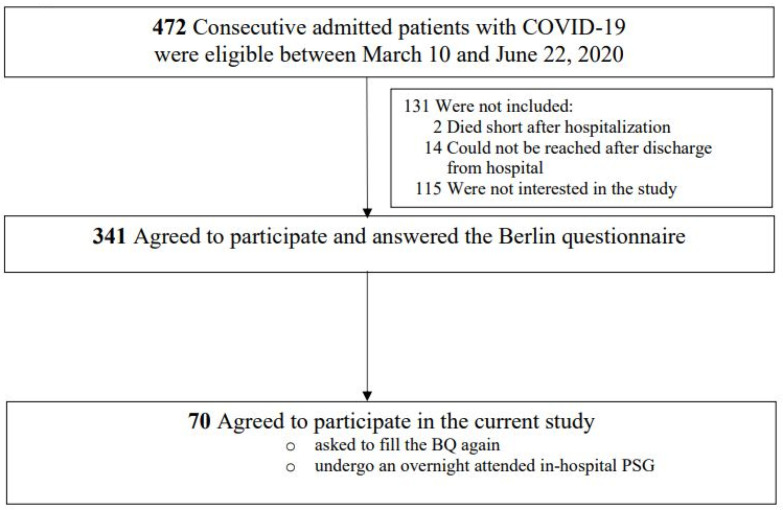
Flow-chart of the study population.

The current study recruited participants who were diagnosed with a COVID-19 infection at Koç University Hospital and Koç Healthcare American Hospital between 10 March and 22 June 2020, and who completed the BQ (Appendix A) in our initial OSACOVID study [9]. All previous participants were invited to join the current validation study. They were asked to fill the BQ again and undergo an overnight attended in-hospital PSG (Appendix B, Figure A2). Demographic data, comorbidities, questionnaires, and PSG findings were collected.

The Koç University Committee on Human Research approved the study protocol (approval no. 2021.231.IRB2.049; 6 May 2021), and a written informed consent was obtained from all participants. The initial OSACOVID-19 study was registered with the ClinicalTrials.gov NCT04363333. For details, see the online supplement. The manuscript was prepared according to the STARD (standards for reporting diagnostic accuracy studies) 2015 guidelines.

### 2.2. Data Collection and Definitions

**Figure A3:**
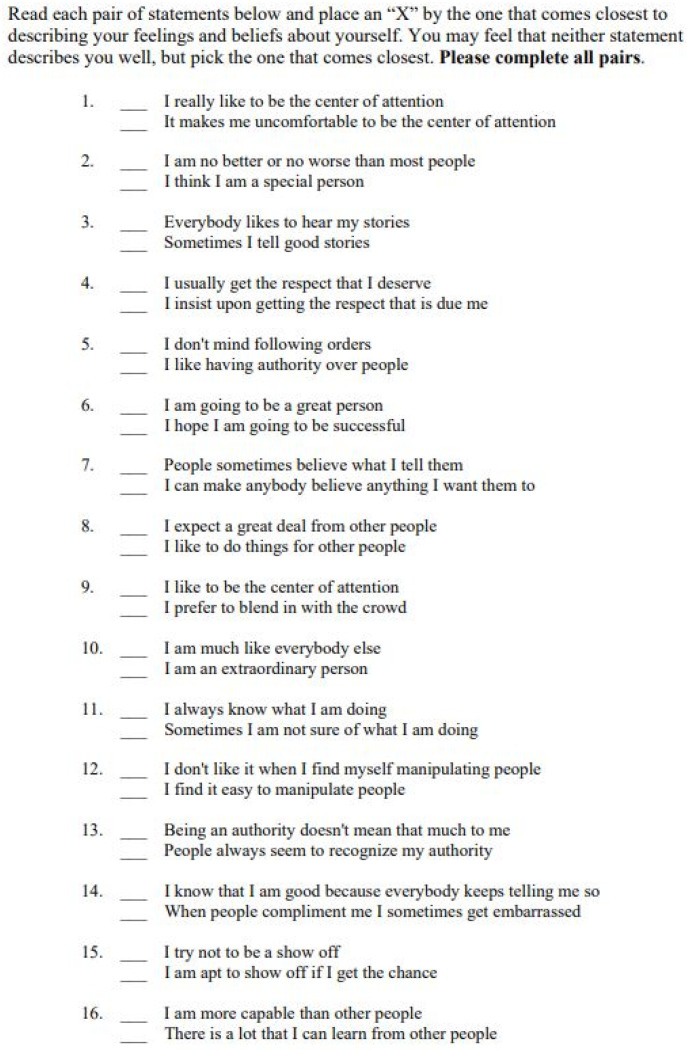
Narcissistic personality inventory.

As previously described [9], all COVID-19 cases were confirmed by positive polymerase chain reaction testing of nasopharyngeal specimens and/or clinical symptoms and radiologic findings suggestive of COVID-19 pneumonia. In addition to the BQ, each participant filled out Narcissistic Personality Inventory (Appendix C, Figure A3) and a questionnaire regarding sleep habits as well as sleep-related symptoms, which were used in clinical routines. Demographics, comorbidities, as well as physical examination findings, including neck, hip, and waist circumference, were documented. Obesity was defined as a BMI of at least 30 kg/m^2^ [12]. The participants’ responses to STOP-Bang Questionnaire (Appendix D) were adopted from the BQ responses and the physical examination findings. 

### 2.3. Modified Berlin Questionnaire

The mBQ comprised 3 subcategories from the BQ: Subcategory 1 included two items from the BQ, which are snoring intensity (item 2) and snoring frequency (item 3). When the answers were “louder than talking or very loud” for the item 2 and/or 3–4 times a week or nearly every day for the item 3, the subcategory provided a positive score. Subcategory 2 contained an item which is asking about witnessed apneas (item 5) and provided a positive score when the response was “3–4 times a week” or “nearly every day”. The last subcategory consisted of two items from the BQ, which are questions regarding tiredness in the morning (item 6) and tiredness during daytime (item 7). A positive score was provided when the response was “3–4 times a week” or “nearly every day” for item 6 and/or item 7. As previously described elsewhere [9], the participants were categorized as having high-risk OSA when they scored positive on 2 or more subcategories.

### 2.4. Sleep Measurements

In the current study, we used a full-night polysomnography (PSG) (NOX-A1 system; Nox Medical Inc., Reykjavik, Iceland) at the sleep laboratory of the Koc University Hospital. The attended PSG included EEG, EOG, chin and leg electromyograms, snoring intensity, nasal airflow, thoraco-abdominal and leg movements, body position, heart rate, SpO2 as well as video recording. Sleep stages and arousals were scored based on 30-s epochs in accordance with The AASM Manual for the Scoring of Sleep and Associated Events 2.5 [13]. Apnea was defined as an almost complete (≥90%) cessation of airflow, and hypopnea was defined as a decrease in nasal pressure amplitude of ≥30% and/or thoraco-abdominal movement ≥ 30% for ≥10 s if there was a significant oxyhemoglobin desaturation (reduction by ≥3% from the immediately preceding baseline value), and/or an arousal, according to the latest recommendations of the AASM [14]. Furthermore, the total number of significant desaturations was scored, and the oxygen desaturation index (ODI) was calculated as the number of significant desaturations per hour of total sleep time. Minimum SpO_2_ and time spent below 90% SpO_2_ (TS90%) values were also recorded. OSA was defined as an AHI ≥ 15 events/h of the total sleep time, based on the latest International Classification of Sleep Disorders-III [14] when OSA-related symptoms are absent. All PSG recordings were manually scored in a mixed order by a certified sleep technician under the supervision of YP blinded to the mBQ categorizations.

### 2.5. Sample Size Estimation

In validity and reliability studies, it is recommended to include minimum 5 and maximum 10 individuals per number of questions that make up the scale [15]. Since the mBQ consisted of 5 items from the BQ, the number of participants to be included was calculated as minimum 25 and maximum 50. In order to increase the power of the study as well as reproducibility of the results, we included 70 adults with a history of COVID-19 infection. 

### 2.6. Statistics

Anthropometric characteristics and PSG findings of the study population as well as the high- and low-risk OSA groups were summarized as mean with standard deviation or median with 25th and 75th percentile for the continuous variables, and as counts with percentages for the categorical variables. Shapiro Wilk test was used for normality assumption. Comparison between the low- and high-risk OSA groups were performed using the Student’s *t*-test or Mann–Whitney rank sum test for the continuous variables, and χ^2^ test or Fisher’s exact test for categorical variables. Overnight PSG findings were used to validate the mBQ. The diagnostic parameters of the mBQ were computed across different AHI cut-offs including diagnostic odds ratio, disease prevalence, sensitivity, specificity, negative likelihood ratio, negative predictive value, positive likelihood ratio, positive predictive value, and accuracy. The area under receiver-operating characteristic (ROC) curve analysis was used to assess the association between AHI on the PSG and the mBQ results (low and high risk) and to predict the best AHI cut-off value. Coefficients between 0.70 and 0.79 are generally regarded as acceptable; between 0.80 and 0.89 are good; between 0.90 and 1.00 are considered excellent [15]. To assess the content validity, five professionals were asked to rate each item of the mBQ and the new scoring system. For each item, a four-point scale was used including (A) Not Relevant, (B) Somewhat Relevant, (C) Quite Relevant, and (D) Highly Relevant. The content validity index (CVI) score was computed as follows: CVI = the number of experts who select C or D/the total number of experts [16]. The items over 0.8 in the CVI were considered as significant [16]. A principal component analysis was conducted to evaluate dimensionality of the mBQ to assess the construct validity. The Kaiser Meyer Olkin (KMO) test was used to evaluate the sampling adequacy, and the Bartlett’s sphericity test was conducted to measure homogeneity of variance. Convergent validity was assessed as a measure of agreement between the STOP Bang questionnaire, and mBQ by using the Cohen’s Kappa value. Moreover, discriminant validity was evaluated regarding Pearson correlation coefficients between the total scores of mBQ and the NPI. The accepted significance level for all tests was set as 5%, and statistical analyses were performed using IBM SPSS 26.0 for Windows SPSS Inc., Chicago, IL, USA.

## 3. Results

### 3.1. Baseline Characteristics of the Study Population

A total of 70 patients (mean age 54.1 (SD = 12.3) years; 72.9% males) were included in the current study. The median BMI was 29.1 (27.1–31.4) kg/m^2^, and 43% of the entire population were obese. The median ESS score was 5.0 (2.0–8.3), and 88.6% of the cohort had an ESS score < 11.

### 3.2. Categorization of the Study Population Based on the Subcategories of the mBQ

**Figure 1:**
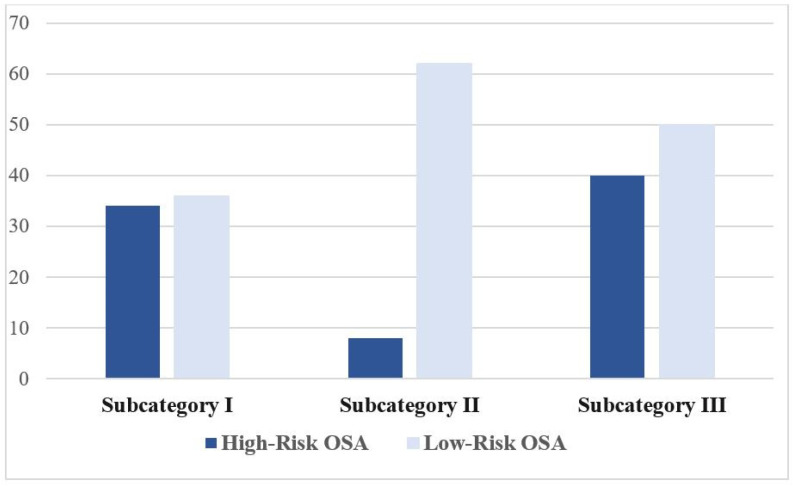
Distribution of the participants’ responses on the subcategories of the mBQ. Definition of abbreviations: mBQ, modified Berlin questionnaire; OSA, obstructive sleep apnea.

As illustrated in Figure 1, out of 70 participants, 27 (38.6%) were categorized as having a high risk of OSA based on the mBQ. Overall, 34 cases (48.6%) were categorized as positive on snoring intensity and/or snoring frequency (Subcategory I), 8 cases (11.4%) on witnessed apneas (Subcategory II), and 40 cases (57.1%) on the tiredness in the morning and/or during the whole day (Subcategory III). 

### 3.3. Baseline Characteristic of the High vs. Low Risk OSA Groups

The baseline characteristics of the participants in the high- and low-risk OSA groups are demonstrated in Table 1. The patients in the high-risk OSA group were older and had higher BMI than the participants in the low-risk OSA group. Obesity, current smoking, alcohol consumption, family history for snoring as well as known OSA diagnosis were similar in both groups. Comorbidities did not differ between groups. The median time between the initial mBQ during the acute COVID-19 infection and the second mBQ for the PSG validation was 406 days (IQR 379–475 days).

### 3.4. PSG Results of the High vs. Low Risk OSA Groups

The PSG results of the participants across two study groups are presented in Table 2. The participants in the high-risk OSA group had significantly worse sleep architecture in terms of longer sleep latency, lesser sleep efficiency, more frequent and longer awakenings after sleep onset, and a lower proportion of slow wave sleep than the sleep architecture in the low-risk OSA group. As expected, significant between-group differences were observed regarding the number of obstructive events per hour as well as the severity of OSA applying different cut-off values of AHI. Thus, more frequent obstructive events during REM and non-REM sleep periods as well as in supine position were observed among the high-risk OSA group. Consequently, the median ODI value was significantly higher than the ODI in the low-risk OSA group. The average and nadir SpO_2_ levels were lower, and the time spent below 90% SpO_2_ was significantly longer in the high-risk OSA group compared to those among the patients with low-risk OSA. 

### 3.5. Diagnostic Utility of the mBQ vs. BQ

**Figure 2:**
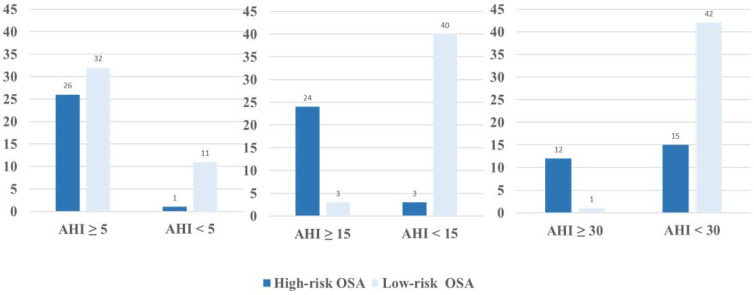
The number of the patients based on the mBQ vs. PSG results. Definition of abbreviations: AHI, apnea−hypopnea index, OSA, obstructive sleep apnea.

The diagnostic performance of the mBQ compared to the PSG results has been presented in Figure 2. Using the AHI cut-off of 5 events/h, 58 patients were categorized as having OSA based on the PSG. Out of these 58, 26 (96.3%) patients were correctly identified as having high-risk OSA by mBQ while 38 (95.0%) patients as high-risk OSA based on the BQ. Similarly, 27 cases were positive on the PSG using the AHI cut-off 15 events/h, of whom 24 (88.9%) were correctly identified by mBQ and 22 (55.0%) of them by the BQ. Corresponding values for the mBQ vs. the BQ were 12 (44.4%) vs. 11 (27.5%) out of 13 positive cases on PSG applying the AHI 30 events/h thresholds, respectively.

The predictive values of the mBQ as well as BQ were calculated at various AHI cut-offs and presented in Table 3. The diagnostic odds ratio was the highest when the AHI threshold was 15 events/h for the mBQ. At this threshold, the mBQ had a sensitivity of 90.5%, a specificity of 89.6%, a positive predictive value of 86.4%, a negative predictive value of 92.9% and an accuracy of 90%. Corresponding values for the BQ were 65.5%, 83.3%, 95.0%, and 33.3%, respectively, using the AHI thresholds 5 events/h with the highest diagnostic odds ratio.

Additional analysis testing AHI cut-off 10 events/h lower sensitivity (72.7 [54.5–86.7]%) and specificity (91.9 [78.1–98.3]%) than the values for AHI cut-off 15 events/h, and the accuracy was also lower (82.9 [72.0–90.8]%). When testing the AHI cut-off 20 events/h, the sensitivity was higher (95.0 [75.1–99.9]%) but the specificity (84.0 [70.1–92.8]%) and the accuracy (87.1 [77.0–94.0]%) were lower than the values for AHI cut-off 15 events/h.

**Figure 3:**
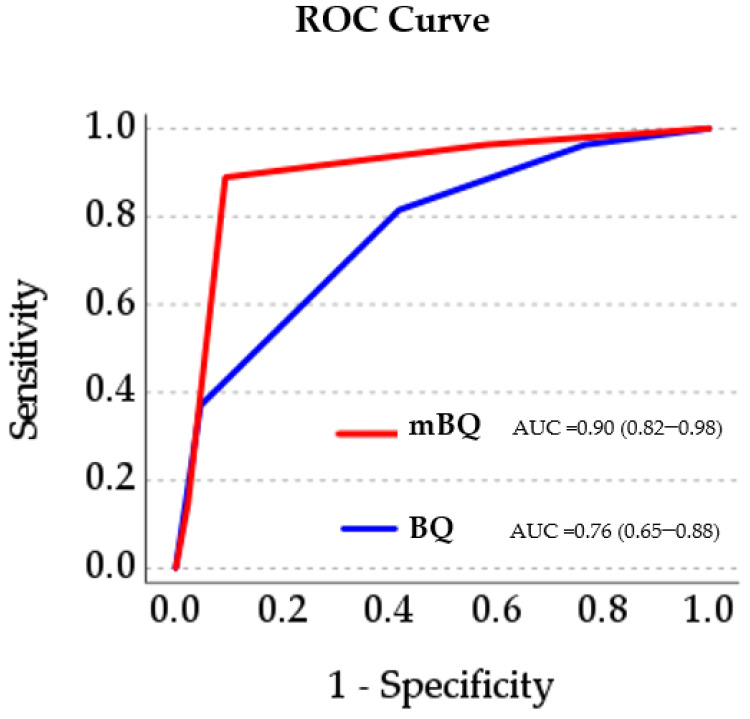
ROC curve of the association between the total scores of mBQ vs. BQ as continuous variables and OSA using AHI ≥ 15/h cut-off. Abbreviations: AHI, apnea−hypopnea index; AUC, area under curve; mBQ, modified Berlin questionnaire; ROC, receiver operating characteristic.

Figure 3 illustrates the ROC curve of the association between the total scores of mBQ as well as the BQ (number of positive subscales as continuous variables) and OSA classification using the cut-off AHI ≥ 15 events/h. For the mBQ, the area under the curve was 0.90 (95% CI 0.82–0.98) confirming a very good performance. Corresponding values for the BQ revealed lower performance results. 

### 3.6. Principal Component Analysis 

A principal component analysis was conducted on the five items with orthogonal rotation (VARIMAX). The Kaiser-Meyer Olkin measure verified that the sampling adequacy was mediocre (0.56) and Bartlett’s test of sphericity result was significant (*p* < 0.001). According to Kaiser’s recommendation [17], these results are barely acceptable to run the factor analysis.

The analysis showed that component 1 (Subcategory I; snoring intensity and frequency) and component 2 (Subcategory III; morning and daytime tiredness) had eigenvalues over of 1 and component 3 (Subcategory II; witnessed apneas) had an eigenvalue below 1 in combination with explained 91% of the variance (Table 4). The component matrix after rotation is presented in Table 4. The items snoring patterns/frequency and witnessed apneas clustered on the component 1, and the items morning and daytime tiredness clustered on the component 2 while the item witnessed apneas clustered on the component 3.

### 3.7. Reliability and Content, Convergent and Discriminant Validities of the mBQ

#### 3.7.1. Reliability of the mBQ

**Figure 4:**
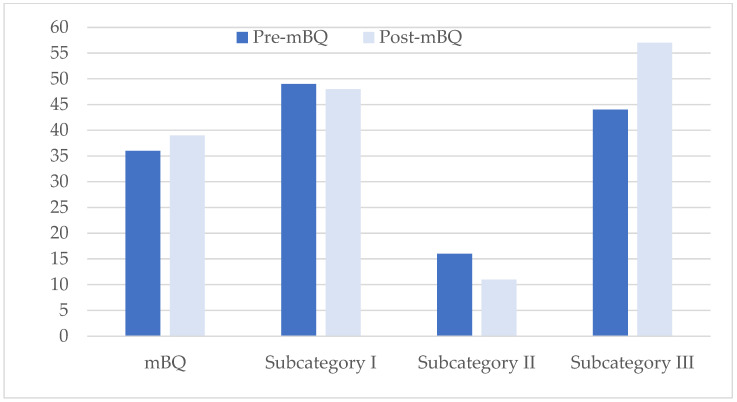
Proportions of the participants who were categorized as high-risk OSA on the first test and retest occasions.

As illustrated in Figure 4, the proportion of participants with high-risk OSA on the first test (36%) was similar to proportion of the high-risk OSA patients on the retest. Corresponding values for the subcategories were 49% vs. 48% for Subcategory I and 16% vs. 11% for Subcategory II. There was an increase in the proportion of patients with a high-risk OSA on Subcategory III from 44% in the first test to 57% in the retest. The agreement between pre-test and retest was calculated as 0.43 (*p* = 0.002), which indicates a fair agreement between two test results. There was no significant difference between test and re-test scores, which indicates similarity of the two test results (Mc Nemar nonparametric test results).

#### 3.7.2. Content, Convergent and Discriminant Validities of the mBQ

Table A1 presents the content validity ratings of the five experts evaluating each item of the mBQ. The items regarding snoring frequency and morning tiredness showed excellent content validity (I-CVI = 1.0) while content validity for the snoring intensity, witnessed apneas and daytime tiredness can be evaluated as appropriate (I-CVI ≥ 0.79) [16]. Regarding the convergent validity of the mBQ, Cohen’s Kappa value as a measure of agreement calculated as 0.43 (*p* = 0.01) indicates moderate agreement between mBQ and STOP-Bang questionnaire. Furthermore, Pearson correlation coefficients between the total scores of mBQ and NPI was 0.11, which indicates there was a weak correlation between scores of the two questionnaires.

## 4. Discussion

The main finding of the current study is that the mBQ has a good predictive ability to detect OSA, defined as an AHI ≥ 15 events/h, in a clinical adult population with a history of COVID-19 infection. The accuracy was high (91.4%) with excellent sensitivity and specificity (88.9% and 93.0%, respectively). The positive and negative predictive values of the test was also high (88.9% and 93.0%, respectively).

The mBQ was also tested for the content validity, which was examined by five professionals in the Sleep Medicine field through the non-face-to-face approach. The experts judged all items as good to excellent, indicating that the mBQ focusing on the subcategories has achieved a satisfactory level of the content validity. 

The construct validity applying a principal component analysis with a three-factor extraction out of the five items revealed that the eigenvalues of Factor 1 and Factor 2 were over 1 whereas Factor 3 had an eigenvalue below 1. The lower eigenvalue for Factor 3 might be due to the fact that it included only 1 item, and that the number of participants who reported witnessed apneas was low (%11.4). Even if the items were related to each other, positive responses to and the proportion of each item differed between the participants.

The test–retest reliability was low, which might be due to long time interval between the two tests. The signs of airway obstruction (snoring and witnessed apneas) seemed to be more common (not significant) during the first occasion with acute upper airway infection whereas tiredness as a sign of post-COVID syndrome was more prevalent at the follow-up survey administration. Since the current study did not aim to confirm the first mBQ results at the acute COVID-19 infection period but to validate the retest mBQ answers when PSG was conducted, the weak correlation between the first mBQ and the retest mBQ may not necessarily mean a weakness of the current protocol.

The convergent validity was confirmed by a moderate agreement between the mBQ and the STOP-Bang questionnaires regarding the classification of participants as high-risk vs. low-risk OSA. Moreover, there was a strong linear correlation between the total scores of the mBQ and the STOP-Bang questionnaires, supporting the use of mBQ as a good alternative screening tool for the OSA diagnosis. 

A strong discriminant validity was confirmed by the weak correlation between the mBQ and the NPI scores.

### 4.1. The Utility of mBQ across the Different AHI Thresholds

#### 4.1.1. AHI ≥ 5 events/h

The definition of OSA is arbitrary, and it has been recommended that an AHI ≥ 5 events/h would be appropriate to perform a validation study in patients at high-risk OSA in epidemiological studies [17]. It has been suggested that the BQ is useful as a clinical screening test and epidemiological tool in the sleep clinic population. Adopting more consistent methodological definitions and focusing more on the general population and specific clinical populations to determine its usefulness as a clinical or epidemiological screening tool are also recommended [18]. Netzer et al., who conducted the first validation study of the BQ with HSAT in a primary care population, reported sensitivity and specificity of 86% and 77%, respectively, based on the AHI cut-off 5 events/h [19]. Later studies using PSG have reported sensitivity and specificity as 69% and 83% in a general population [20] and 76% and 40% in the primary care setting [21], respectively. Another validation study in a general population in India showed higher sensitivity (86%) and specificity rates (95%) when the researchers modified the BQ by excluding item 9 (Have you ever nodded off or fallen asleep while driving a vehicle) and decreasing the BMI threshold from 30 to 25 kg/m^2^ [22]. Comparing to those studies, our mBQ results showed poorer sensitivity (44.8%) but similar specificity (91.7%) for the AHI cut-off 5. The poor sensitivity of the mBQ at this cut-off level might be due to the fact that the mBQ was validated in a clinical cohort with a history of COVID-19 infection, not in a general or primary care population. It may also mean that this cut-off level might result in an increased number of false negative cases in clinical cohorts. On the other hand, the number of false positive cases was minimized in our cohort, which reflects the high specificity of the mBQ at this AHI threshold. 

As is known, the sensitivity and specificity are usually inversely related with each other, and the high specificity often comes with a reduced sensitivity. When the cost of a gold standard is very expensive, false positive rates should be minimized. Thus, the screening tool applying the AHI threshold 5 shows better performance to identify a patient who would unnecessarily undergo PSG. On the other hand, the low sensitivity would culminate in more PSG investigations due to increased number of false positive cases.

#### 4.1.2. AHI ≥ 15 events/h

The international task force on standardization of definition of sleep-related breathing disorders recommends AHI 15 events/h as cut-off in the absence of symptoms and medical or psychiatric disorders [23,24]. This threshold has been more frequently used for the detection of clinically relevant OSA as well as in validation studies. Applying the AHI 15 cut-off, the predictive values of the BQ in sleep clinical population studies ranged from 58.5% to 95.5% for sensitivity and from 16.2% to 61.0% for specificity. A recent meta-analysis including sleep clinic studies concluded that the BQ had a moderate to high sensitivity but low specificity to detect clinically relevant OSA in different clinic populations such as cardio/cerebrovascular disease, surgical patients, patients with chronic obstructive pulmonary disease, and individuals with resistant hypertension [25]. Our results, showing a sensitivity of 88.9% and a specificity of 93.0%, are remarkably in contrast with the aforementioned studies. The differences may be attributable to the main rationale of our study, namely, the modification of the BQ per se by excluding obesity and hypertension as well as restructuring the subcategories focused on snoring frequency/intensity, witnessed apneas, and morning/daytime tiredness [9]. The exclusion of hypertension and obesity may have minimized the number of false positive cases, which might have increased the specificity of the mBQ. Similarly, focusing on the clinically important symptoms as subcategories might have resulted in a decreased number of false negative cases, and thus, the sensitivity rate might have been improved. 

#### 4.1.3. AHI ≥ 30 events/h

In the literature, the diagnostic performance of the BQ to predict severe OSA (AHI ≥ 30 events/h) was also examined in sleep clinic cohorts reporting predictive values of the BQ with a great variation, ranging from 30.8% to 97.3% for sensitivity and 10.7% to 80.0% for specificity [25]. A small number of validation studies addressing the AHI threshold of 30 as well as the wide range reported in those studies make it difficult to draw proper conclusions in this context. Our results demonstrated a high sensitivity (92.3%), but the specificity was moderate (73.7%) to predict severe OSA. Previously, it has been suggested that the ability of the BQ to predict the occurrence of OSA increases with an elevated AHI [20]. However, those results were not supported by the latter studies [23,26,27,28]. Similarly, our results showed no linear relationship between the predictive performance of the mBQ and OSA severity in terms of the AHI thresholds 5, 15, and 30, respectively.

### 4.2. Methodological Considerations in the BQ Validation Studies

The observed variability in the previous validation studies might be mainly attributable to use of different overnight sleep recordings (PSG vs. HSAT) [19,22,29]. The PSG is the gold standard for the diagnosis of OSA. However, given the high cost for the PSG investigations, it is difficult to conduct a validation study in a general population using PSG. Apart from the differences regarding the sleep recording devices used in the validation studies, the changes in scoring criteria for hypopneas over time (for example, 3% vs. 4% thresholds of accompanying desaturations and/or arousals) may also explain some of the variations in the previous validation studies [25]. 

### 4.3. Association of OSA with a History of COVID-19 Infection

Literature regarding the true occurrence of OSA in patients with a history of COVID-19 infection is scarce given that objective sleep studies with PSG was not feasible during the pandemic. In two retrospective cohort studies, a known OSA diagnosis was reported among 9.5% and 12.3% of cases [3,8]. Cade et al. reported that COVID-19 patients with a known OSA diagnosis had a higher mortality rate compared to the control subjects [8]. It has been argued that the COVID-19 pandemic is globally urging the need for new approaches beyond the PSG requirement for the management of OSA cases [30,31]. In this context, our results are, indeed, promising.

### 4.4. Potential Mechanisms Linking OSA with a History of COVID-19 Infection

In our initial study, the estimated occurrence of OSA was 38% out of 320, based on the BQ, among the patients during the acute phases of COVID-19 infection. This occurrence rate was much higher than that previously reported in a nationwide study in Turkey, 14% among 5021 adults [10]. However, as discussed previously [9], the items of Category 3 (obesity and hypertension) of the BQ complicate the statistical adjustments when addressing the prognosis of patients with COVID-19. Therefore, a modified scoring based on the snoring patterns (louder and/or frequent snoring), breathing pauses, and morning and/or daytime tiredness/sleepiness was the main rationale of our initial study. The prevalence of modified high-risk OSA was estimated to be around 22% when obesity and hypertension were not considered.

The significant predictive effects of the snoring patterns, especially louder snoring, in our initial study, were indeed notable. Recently, the self-reported snoring patterns predicted stroke incidence in high-risk patients with OSA in the SAVE (sleep apnea cardiovascular endpoints) cohort [32]. The authors also highlighted the controversy over whether snoring is a symptom or a surrogate marker of OSA. Furthermore, it was debated whether the adverse cardiovascular effects are driven by obstructive events and hypoxemia, or from the trauma of vibrations owing to snoring per se [32]. In line with these approaches, our findings provide further insights into the relationship between OSA and COVID-19 infection. As also discussed in our previous article, the individuals with louder snoring might be more prone to be infected by COVID-19. They may also develop pneumonia with a poorer prognosis because of the trauma around the upper airway muscles caused by the vibrations. It may also be argued that the COVID-19 infection per se may increase the collapsibility of the upper airway muscles, which may trigger or worsen OSA. Thus, the association between OSA and COVID-19 onset as well as prognosis might be bidirectional [9].

Other potential mechanisms linking OSA to an increased risk for predisposition to COVID-19 infection and poor outcomes have been discussed recently [5,6,31]. OSA, particularly with concomitant obesity, could potentially worsen hypoxemia and the cytokine storm that occurs in patients with COVID-19 [6]. Furthermore, myocardial injury involving the angiotensin-converting enzyme-2 signaling pathways, systemic inflammation, and hypercoagulability should be considered in this context [5]. OSA could be a trigger of COVID-19 infection, and once the disease has occurred, it could contribute to worsening of prognosis, especially among the cases with hypertension and diabetes. Moreover, many patients with COVID-19 suffer pulmonary fibrosis, which itself is associated with the future development of OSA [31]. Hence, it is clear that the COVID-19 pandemic has had a major effect on the treatment management and diagnosis of OSA, and it is crucial to explore new diagnosis and treatment pathways for these individuals, who are at a high risk of increased morbidity and mortality [31].

### 4.5. Strengths and Limitations of the Study

The strengths of the current study include (1) the use of PSG as the diagnostic tool for OSA to validate the mBQ; (2) being the first validation study of the mBQ in a cohort with a history of COVID-19 infection. 

Certain limitations should also be acknowledged. The gender distribution in the first study among 320 COVID-infected patients was almost equal, 54% men vs. 46% women, but there was a higher proportion of men in the current study (73%), which may reflect that the patients who participated in the current overnight PSG study were more likely to be the ones who had sleep-related problems, and thus, a sample selection bias cannot be excluded. The gender imbalance also limits the generalizability of the current findings to the general population. 

### 4.6. Clinical Implications and Future Perspectives

Due to the increasing awareness about the burden of OSA in terms of cost and adverse health consequences, there is a growing interest in early diagnosis and treatment of OSA. In that context, the BQ has been one of the widely used screening tools for OSA. A recent systematic review and meta-analysis [18] concluded that the BQ has a modest to high sensitivity but low specificity to detect OSA in sleep clinic patients. There was limited evidence suggesting the BQ as a useful screening tool for those with cardio/cerebrovascular disease, as well as for general, primary care, and surgical populations. Our modified BQ had a much better sensitivity and specificity than BQ in this cohort of patients with a history of COVID-19 infection. Since the confounding effects of obesity and hypertension are eliminated, we may also suggest that the mBQ can be used not only as a screening tool for high-risk OSA but also as a prognostic survey in clinical cohorts.

Based on our mBQ scores, 39% of the current study population was classified as having high-risk OSA, and the majority of those patients were cumulated on the snoring intensity and frequency as well as morning and daytime tiredness. Given the anthropomorphic and clinical characteristics of the study participants who are more likely to be a primary care cohort rather than a sleep clinic cohort, these symptoms might be disregarded by the general practitioners who take care of individuals with a history of COVID-19 infection. Thus, the mBQ seems to be an ideal screening tool for OSA in the primary care setting.

## 5. Conclusions

The mBQ is a practical, feasible, and cost-effective method to predict OSA in adults with a history of COVID-19 infection, and has a good level of diagnostic sensitivity, specificity, and accuracy. It can be used in the clinical management of cases with high-risk OSA in hospitals with long waiting lists for PSG and to eliminate unnecessary PSG investigations for adults with low-risk OSA. The utility of the mBQ in general as well as other clinical populations’ needs to be evaluated in future studies.

## Figures and Tables

**Table 1:** Demographics characteristics of the high and low risk OSA groups.

	High-Risk OSA (*n* = 27)	Low-Risk OSA (*n* = 43)	*p*
Age, years *	59.04 (9.2)	51.07 (13.22)	0.01
Male Sex, (%)	23 (85.2)	28 (65.1)	0.66
Married	25 (92.6)	32 (78.0)	0.11
BMI, kg/m^2^ *	30.4 (28.1–32.8)	27.9 (26.3–30.9)	0.04
Obesity, (%)	15 (55.6)	15 (34.9)	0.08
ESS	5.0 (3.0–10.0)	5.0 (2.0–8.0)	0.84
Current Smoker	2 (7.4)	4 (9.3)	0.74
Alcohol consumption	7 (26.9)	9 (22.0)	0.43
Allergy	9 (36.0)	8 (19.0)	0.12
Asthma/COPD	4 (15.4)	6 (14.3)	0.90
Hypertension	7 (26.9)	13 (31.0)	0.72
Angina pectoris	3 (11.5)	6 (14.0)	0.77
AMI	1 (3.7)	2 (4.7)	0.85
PCI/CABG	1 (3.7)	4 (9.3)	0.38
Cardiac failure	2 (7.7)	3 (7.0)	0.91
Arrhythmia	4 (14.8)	6 (14.0)	0.92
Stroke	0 (0)	2 (4.7)	0.27
Hyperlipidemia	9 (36.0)	12 (27.9)	0.50
Diabetes Mellitus	7 (28.0)	10 (23.3)	0.66
Hypothyroidism	4 (14.8)	10 (23.3)	0.39
Neurological Disorder	2 (7.4)	3 (7.0)	0.95
Muscle Disorder	1 (3.7)	1 (2.4)	0.75
Psychiatric Disorder	1 (3.7)	5 (11.6)	0.25

Continuous data are presented as median with min–max values. Categorical data are presented as count with percentage. The Student’s *t*-test or Mann–Whitney Rank Sum test for the continuous variables, and χ^2^ test or Fisher’s exact test for categorical variables. Abbreviations: AMI, acute myocardial infarction; BMI, body mass index; CABG, coronary artery bypass grafting; COPD, chronic obstructive pulmonary disease; ESS, Epworth sleepiness scale; OSA, obstructive sleep apnea; PCI, percutaneous coronary intervention. * *p* < 0.005 at the significant level.

**Table 2:** PSG results of the high and low risk OSA groups.

	High-Risk OSA (*n* = 27)	Low-Risk OSA (*n* = 43)	*p*
TST, min	384.0 (47.2)	393.8 (52.9)	0.34
SE, % of TST *	79.1 (8.5)	83.0 (9.9)	0.048
Sleep Latency, min	37.1 (21.8–57.70)	29.3 (15.1–52.4)	0.201
WASO, min *	62.5 (36.5–84.8)	33.1 (20.5–53.5)	0.009
SWS duration, min	79.8 (27.7)	106.3 (38.3)	0.002
SWS, % of TST *	20.4 (13.9–25.7)	28.5 (20.9–32.3)	<0.001
REM duration, min	68.5 (29.1)	74.1 (26.2)	0.223
REM, % of TST	17.2 (6.9)	18.4 (5.7)	0.197
REM latency, min	134.6 (121.0–228.2)	152.0 (99.5–192.5)	0.722
AHI, events/h *	22.50 (17.70–35.10)	7.3 (3.7–9.2)	<0.001
AHI REM, events/h *	37.1 (21.0–51.0)	11.2 (2.3–22.4)	<0.001
AHI non-REM, events/h *	24.4 (13.3–32.6)	4.7 (2.7–8.5)	<0.001
Supine position, min	185.6 (95.3–265.4)	156.8 (72.5–241.3)	0.334
Supine position, % of TST	45.6 (23.8–61.4)	40.4 (18.1–58.1)	0.264
AHI supine, events/h *	39.5 (26.3–57.0)	13.4 (7.0–22.7)	<0.001
ODI, events/h *	18.7 (14.6–28.1)	6.1 (2.7–6.9)	<0.001
Average SpO_2_ % *	92.5 (1.5)	93.7 (1.7)	0.001
Minimum SpO_2_ % *	81.0 (78.0–83.0)	87.0 (82.0–89.0)	<0.001
SpO_2_ < 90%, min *	16.5(5.0–35.9)	1.1 (0.2–14.8)	<0.001
SpO_2_ < 90%, % of TST *	3.8 (1.1–9.2)	0.3 (0.00–3.7)	<0.001
Average SpO_2_ drop, % *	4.6 (4.0–5.2)	3.7 (3.4–4.1)	<0.001
Heart rate, bpm	63.3 (8.7)	62.5 (7.5)	0.875

Continuous data are presented as mean and standard deviation or median and 25–75% quartiles. The Student’s *t*-test or Mann–Whitney rank sum test for the continuous variables. AHI, apnea−hypopnea index; AMI, acute myocardial infarction; BMI, body mass index; bpm, beat per minute; CABG, coronary artery bypass grafting; COPD, chronic obstructive pulmonary disease; DBP, diastolic blood pressure; ESS, Epworth sleepiness scale; ODI, oxygen desaturation index; OSA, obstructive sleep apnea; PCI, percutaneous coronary intervention; REM, repeat eye movements; SBP, systolic blood pressure; SE, sleep efficiency; SpO2, oxyhemoglobin saturation; SWS, slow wave sleep; TST, total sleep time; WASO, wake after sleep onset. * *p* < 0.005 at the significant level.

**Table 3:** Predictive parameters (values with 95% CI) for the mBQ and the BQ to identify risk of OSA with different AHI cut-offs.

		AHI ≥ 5/h	AHI ≥ 15/h	AHI ≥ 30/h
mBQ	DOR	6.7 (0.8–54.6)	106.7 (19.9–571.4)	33.6 (4.0–280.1)
	Sensitivity, %	44.8 (31.7–58.5)	88.9 (70.8–97.7)	92.3 (63.9–99.8)
	Specificity, %	91.7 (61.5–99.8)	93.02 (80.9–98.7)	73.7 (60.3–84.5)
	PLR	5.4 (0.8–35.9)	12.7 (4.2–38.3)	3.5 (2.2–5.6)
	NLR	0.6 (0.4–0.8)	0.1 (0.04–0.4)	0.1 (0.02–0.7)
	DP, %	82.9 (71.9–90.8)	38.6 (27.2–51.0)	18.6 (10.3–29.7)
	PPV, %	96.3 (79.6–99.4)	88.9 (72.7–96.0)	44.4 (33.5–55.9)
	NPV, %	25.6 (20.5–31.4)	93.0 (82.1–9.5)	97.7 (86.4–99.6)
	Accuracy, %	52.86 (40.6–64.9)	91.4 (82.3–96.8)	77.1 (65.6–86.3)
BQ	DOR	9.5 (1.9–47.6)	6.1 (1.9–19.1)	5.3 (1.1–26.1)
	Sensitivity, %	65.5 (51.9–77.5)	81.4 (61.9–93.7)	84.6 (54.6–98.1)
	Specificity, %	83.3 (51.6–97.9)	58.1 (42.1–72.9)	49.1 (35.6–62.7)
	PLR	3.9 (1.1–14.1)	1.95 (1.4–2.9)	1.7 (1.2–2.4)
	NLR	0.4 (0.3–0.6)	0.3 (0.1–0.7)	0.3 (0.1–1.2)
	DP, %	82.9 (71.9–90.8)	38.6 (27.2–50.9)	18.6 (10.3–29.7)
	PPV, %	95.00 (84.1–98.6)	55.0 (45.1–64.5)	27.5 (21.2–34.9)
	NPV, %	33.3 (24.4–43.6)	83.3 (54.8–77.9)	93.3 (79.2–98.1)
	Accuracy, %	68.6 (56.4–79.2)	67.1 (54.8–77.9)	55.7 (43.3–67.6)

Data are presented as a value with 95% confidence intervals. Abbreviations: DOR, diagnostic odds ratio; DP, disease prevalence; NLR, negative likelihood ratio; NPV, negative predictive value; PLR, positive likelihood ratio; PPV, positive predictive value.

**Table 4:** Lists of the eigenvalues associated with each linear component and the rotated component matrix.

Total Variance Explained						
Component	Initial Eigenvalues			Rotation Sums of Squared Loadings		
	Total	% of Variance	Cumulative %	Total	% of Variance	Cumulative %
1	2.3	46.2	46.2	1.8	36.2	36.2
2	1.6	32.5	78.8	1.7	35.3	71.5
3	0.6	12.6	91.4	0.9	19.9	91.5
4	0.2	5.1	96.6			
5	0.1	3.3	100.0			
**The Rotated Component Matrix**						
		**1**		**2**		**3**
Snoring intensity		**0.91**		0.04		0.23
Snoring frequency		**0.93**		0.06		0.15
Witnessed apneas		0.29		0.07		**0.95**
Morning tiredness		0.08		**0.93**		0.01
Daytime tiredness		0.02		**0.93**		0.09

**Figure A1:**
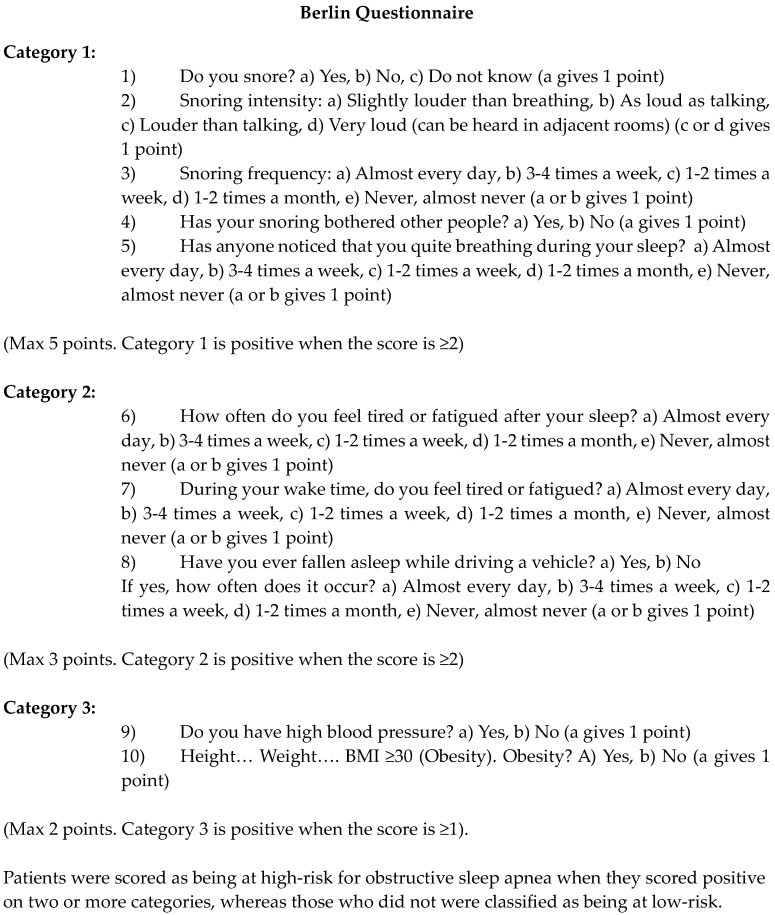
The Berlin questionnaire.

**Figure A4:**
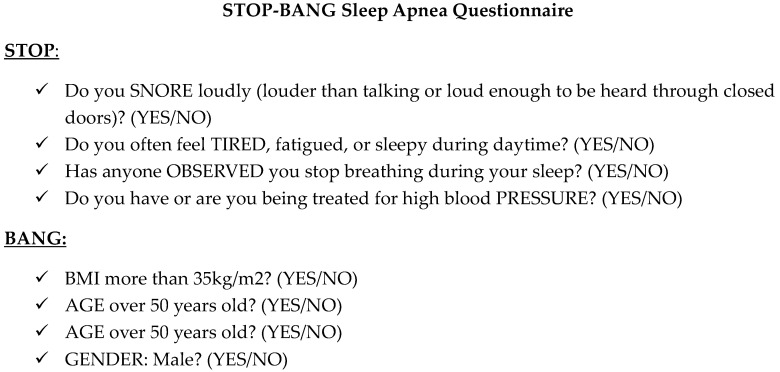
STOP-Bang questionnaire.

## Data Availability

Data collected for the study, including de-identified individual participant data will be made available to others within 6 months after the publication of this article, as will additional related documents (study protocol, statistical analysis plan, and informed consent form), for academic purposes (e.g., meta-analyses), upon request to the corresponding author (yecelik@ku.edu.tr), and with a signed data access agreement.

## Appendix E

**Table A1:** The ratings of five sleep experts for the items of mBQ.

Items of the mBQ	A	B	C	D	CVI
Your snoring is; c. Louder than talking d. can be heard in adjacent rooms		1	3	1	0.8
How often do you snore? a. Nearly every day b. 3–4 times a week			3	2	1
Has anyone noticed that you quit breathing during your sleep? a. Nearly every day b. 3–4 times a week		1	3	1	0.8
How often do you feel tired or fatigued after your sleep? a. Nearly every day b. 3–4 times a week			3	2	1
During your waking time, do you feel tired, fatigued or not up to par? a. Nearly every day b. 3–4 times a week		1	2	2	0.8

Abbreviations; A. not relevant, B. somewhat relevant, C. quite relevant, D. highly relevant.
